# Drone vs. Bird Detection: Deep Learning Algorithms and Results from a Grand Challenge

**DOI:** 10.3390/s21082824

**Published:** 2021-04-16

**Authors:** Angelo Coluccia, Alessio Fascista, Arne Schumann, Lars Sommer, Anastasios Dimou, Dimitrios Zarpalas, Miguel Méndez, David de la Iglesia, Iago González, Jean-Philippe Mercier, Guillaume Gagné, Arka Mitra, Shobha Rajashekar

**Affiliations:** 1 Department of Innovation Engineering, University of Salento, 73100 Lecce, Italy; alessio.fascista@unisalento.it; 2 Fraunhofer Center for Machine Learning, Fraunhofer IOSB, 76131 Karlsruhe, Germany; arne.schumann@iosb.fraunhofer.de (A.S.); lars.sommer@iosb.fraunhofer.de (L.S.); 3 Centre for Research and Technology Hellas, The Visual Computing Lab, Information Technologies Institute, 57001 Thessaloniki, Greece; dimou@iti.gr (A.D.); zarpalas@iti.gr (D.Z.); 4 Gradiant, Galician Research and Development Center in Advanced Telecommunications, 36310 Vigo, Spain; mmendez@gradiant.org (M.M.); diglesia@gradiant.org (D.d.l.I.); igonro@gradiant.org (I.G.); 5 Aerex Avionics Inc., Quebec City, QC G6Z 8G8, Canada; jpmercier87@gmail.com; 6 Defence Research and Development Canada, Quebec City, QC G3J 1X5, Canada; guillaume.gagne@drdc-rddc.gc.ca; 7 Department of Electronics and Electrical Communication Engineering, Kharagpur 721302, India; thearkamitra@iitkgp.ac.in; 8 Displays & Graphics CoE, Aerospace, Honeywell, Los Angeles, CA 65479, USA; shobha.rajashekar@honeywell.com

**Keywords:** drone detection, deep learning, drone vs. bird, automatic recognition, image and video signal processing

## Abstract

Adopting effective techniques to automatically detect and identify small drones is a very compelling need for a number of different stakeholders in both the public and private sectors. This work presents three different original approaches that competed in a grand challenge on the “Drone vs. Bird” detection problem. The goal is to detect one or more drones appearing at some time point in video sequences where birds and other distractor objects may be also present, together with motion in background or foreground. Algorithms should raise an alarm and provide a position estimate only when a drone is present, while not issuing alarms on birds, nor being confused by the rest of the scene. In particular, three original approaches based on different deep learning strategies are proposed and compared on a real-world dataset provided by a consortium of universities and research centers, under the 2020 edition of the Drone vs. Bird Detection Challenge. Results show that there is a range in difficulty among different test sequences, depending on the size and the shape visibility of the drone in the sequence, while sequences recorded by a moving camera and very distant drones are the most challenging ones. The performance comparison reveals that the different approaches perform somewhat complementary, in terms of correct detection rate, false alarm rate, and average precision.

## 1. Introduction

The use of drones, whose origin is in the military domain, has been extended to several application fields including traffic and weather monitoring [1], precision agriculture [2], and many more [3]. With the COVID-19 pandemic, there has been a radical increase in the use of drones for autonomous delivery of essential grocery and medical supplies, but also to enforce social distancing. Nowadays small quadcopters can be easily purchased on the Internet at low prices, which brings unprecedented opportunities but also poses a number of threats in terms of safety, privacy and security [4].

Already from the early days of powered flight, birds have been a concern to aviation safety. Since 1912, bird strikes have caused 47 fatal accidents involving commercial air transport [5]. Since aircraft and birds share the same airspace at lower altitudes, the hazard of bird strikes always exists. The cost of bird strikes to the aviation industry is estimated to be more than one billion euros annually [6]. Similarly, unmanned aerial vehicles (UAVs) bring an important set of challenges to the aviation industry. Despite the restrictions of flying drones near airports, and reserved airspaces to ensure that they do not infringe each others’ area, there are still several challenges involved.

Adopting effective detection and countermeasure techniques to face the rising threat of small drones is a great concern of security agencies and governments today. There is indeed a gap in current surveillance systems, since the small size and fast maneuvers make drones a difficult category of targets to be detected, compared to traditional aircrafts [7,8,9,10,11]. Surveillance and detection technologies based on different modalities are under investigation, with different trade-offs in terms of complexity, maximum coverage in range, and capabilities [12]. Technologies that have been employed include 3D LIDAR sensors, passive radio detectors, video analytics, and passive acoustic sensors [13,14]. All these technologies have pros and cons, if individually considered, and can be effective only under specific operating conditions. Systems implementing an integrated approach that combines the benefits of different technologies have been recently investigated also in Horizon 2020 European research projects SafeShore (http://safeshore.eu, accessed on: 14 April 2021) [15] and ALADDIN (https://aladdin2020.eu, accessed on: 14 April 2021).

Technology can be well utilized in such cases to reduce human effort and provide analytical data and representation. Artificial Intelligence (AI) is one such field that tries to mimic how humans learn and is an active research field. This paper focuses on recent advances on the detection of drones or birds using machine learning based approaches, more specifically deep learning algorithms. The need for such sophisticated methods stems from the recognised difficulty for air traffic controllers to detect and differentiate between birds and drones. Even in computer vision, detecting and classifying drones and birds under different operational conditions is a very challenging task. They are also not easy to be detected at large distances as most object detection models are designed for medium-sized object detection. Convolutional Neural Networks (CNNs) tend to look for meaningful features that can help to classify the images or, in the case of object detection, to draw the boundary boxes enclosing the target of interest. Unfortunately, only limited annotated datasets were available for this purpose.

A Drone vs. Bird Detection Challenge has been launched (https://wosdetc2020.wordpress.com/Dronevs.Bird-detection-challenge/, accessed on: 14 April 2021) in 2017, during the first edition of the International Workshop on Small-Drone Surveillance, Detection and Counteraction Techniques (WOSDETC) [16] organized in Lecce, Italy, as part of the 14th edition of the IEEE International Conference on Advanced Video and Signal based Surveillance (AVSS). A second edition of the challenge has been organized in 2019, again as part of the WOSDETC and held at the 16th edition of AVSS in Taipei, Taiwan. The aim of this challenge is to address the technical issues of discriminating between drones and birds [16,17]. Given their characteristics, in fact, drones can be easily confused with birds, particularly at long distances, which makes the surveillance task even more challenging. The use of video analytics can solve the issue, but effective algorithms are needed that can operate under unfavorable conditions, namely weak contrast, long range, low visibility, etc.

The present paper discusses and compares the most recent advances in this respect, originated from the 3rd edition of the grand challenge held in 2020. The dataset of provided video sequences has been expanding over the years, as it will be described in the following section, and represents today one of the few freely available datasets for research in video-based drone detection. Over the three editions of the challenge, more than one hundred research groups from all over the world have obtained the dataset.

The rest of this paper is organized as follows. Section 2 describes the Drone vs. Bird Detection Challenge 2020, including details on the dataset, evaluation protocol, and participating teams. Section 3 provides a review of the state of the art on drone detection, with emphasis on deep learning approaches, also reviewing the other datasets available in the literature. Section 4, Section 5 and Section 6 are individually devoted to the three best teams that excelled in the 2020 edition of the Drone vs. Bird Detection Challenge, named “Gradiant Team”, “Eagledrone Team”, and “Alexis Team”: the proposed approach, processing, and evaluation results are illustrated for each team. Section 7 reports the performance comparison among the three methods, and finally Section 8 concludes the paper.

## 2. Drone vs. Bird Detection Challenge 2020

In the following section, the Drone vs. Bird Detection Challenge 2020 is described. First, the dataset and its characteristics are introduced, followed by a description of the evaluation protocol. Then, some general information about the participation in the challenge is provided. Note that due to the COVID-19 pandemic, the challenge was not part of the 17th edition of AVSS in Washington DC, USA, which was postponed. Instead, the challenge was organized as a separate virtual event.

### 2.1. Dataset

**Figure 1:**
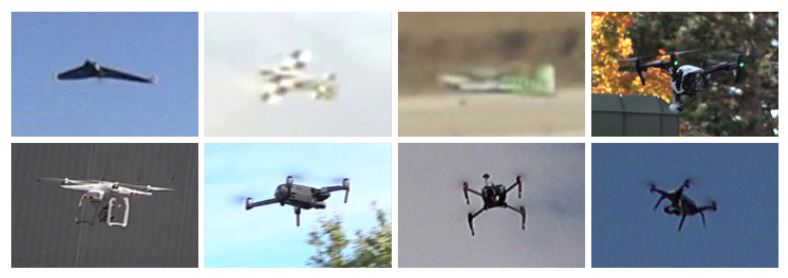
Examples of drone types present in the dataset, i.e., Parrot Disco, 2 custom fix wing drones, DJI Inspire, DJI Phantom, DJI Mavic, DJI Matrice and 3DR Solo Robotics.

The training set for the challenge consists of 77 different videos. The videos comprise 1384 frames on average, while each frame contains on average 1.12 annotated drones. Eleven of these videos originate from the previous installment of the challenge and were collected using MPEG4-coded static cameras by the SafeShore project and by the Fraunhofer IOSB research institute. In 2020, 45 further videos have been contributed by the ALADDIN project in 3 different resolutions, namely 1920 × 1080 @25 fps, 720 × 576 @50 fps, 1280 × 720 @30 fps. The videos have been recorded at two locations with different geo-characteristics. The videos contain 7 types of drones: 3 with fixed wings and 4 rotary ones. The sequences are mostly short in duration (about 1 min each) with the exception of 2 videos that are 5 min long. The remaining 21 training videos were provided by Fraunhofer IOSB and were recorded at different locations in Germany. Such videos include 5 rotary drone types. In total, the videos exhibit 8 different types of drones (see Figure 1).

**Figure 2:**
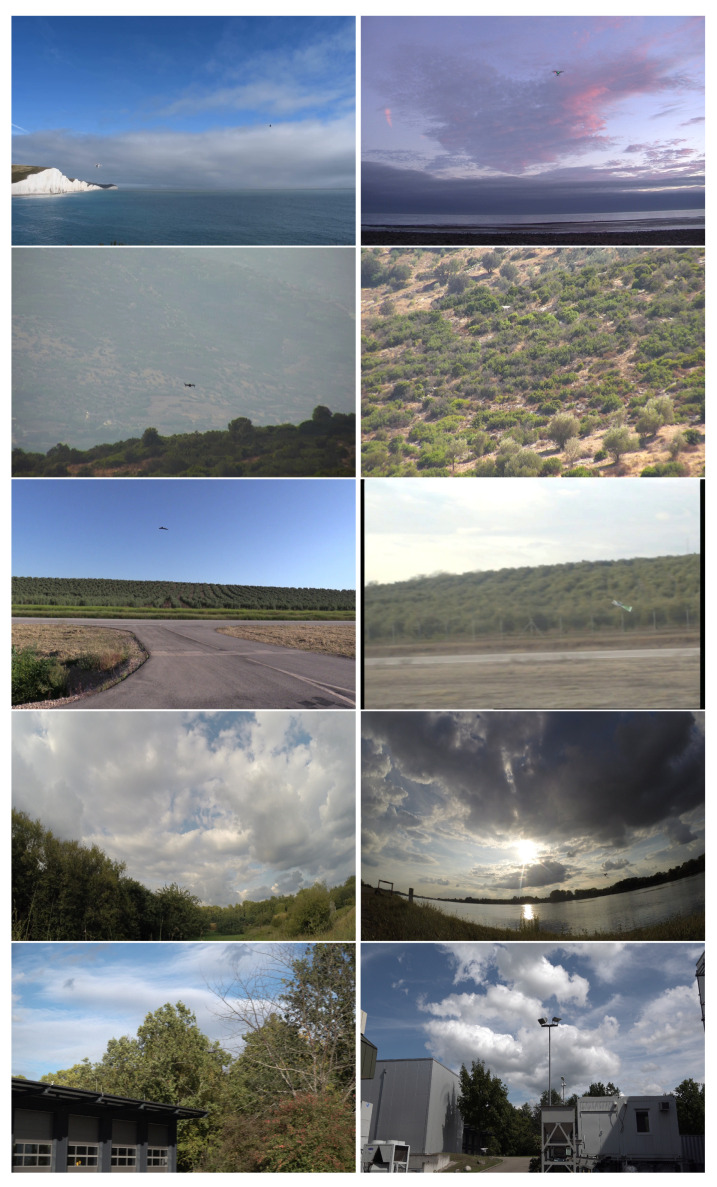
Sample frames extracted from the training videos exhibit the large variability of the dataset.

**Figure 3:**
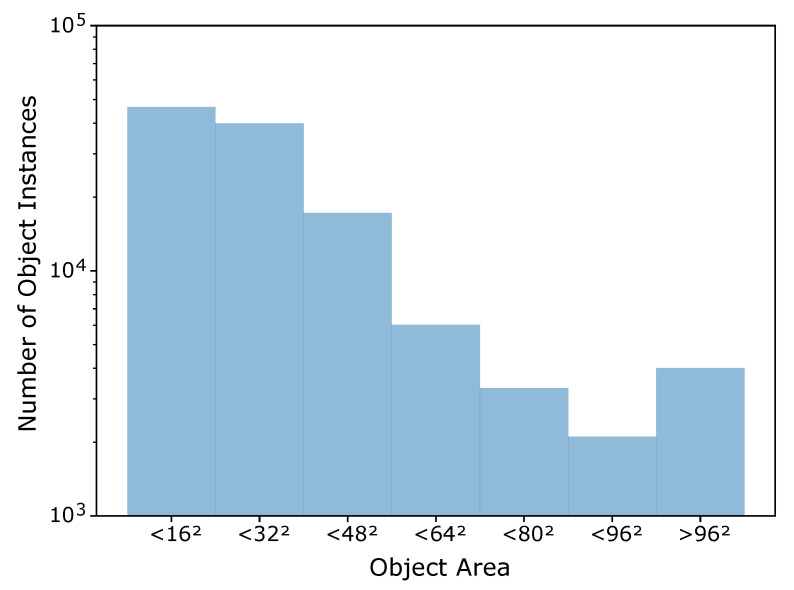
Distribution of drone sizes across the ground truth annotations in the train data.

**Figure 4:**
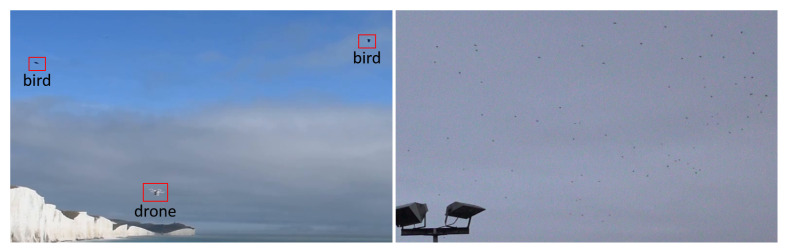
Examples with birds that are the main disturbing objects.

Overall, the training set exhibits a high variability in difficulty, including sequences with sky or vegetation as background, different weather types (cloudy, sunny), direct sun glare and variation in camera characteristics (see Figure 2). Moreover, the distance of the drones from the camera varies strongly across and within the videos, yielding large variations in drone sizes as shown in Figure 3. The drone sizes are in the range between 15 pixels and more than 1,000,000 pixels. The majority of annotated drones exhibits a size less than 16^2^ pixels or in the range between 16^2^ and 32^2^ pixels. The small size of occurring drones makes the detection task very challenging. Many of the videos are recorded with a static camera but also moving camera recordings are included. Each video is associated with a separated annotation file containing the frame number and the bounding box (expressed as $\left[{\mathrm{top}}_{x}\;{\mathrm{top}}_{y}\;\mathrm{width}\;\mathrm{height}\right]$) for the frames in which drones enter the scenes. Birds not individually annotated occur in several of the videos and are the main disturbing object type (see Figure 4).

The challenge test set consists of further 14 videos for which no annotations are provided. The videos contain similar characteristics to the training set. Some of the locations displayed in the test set are also contained in the training set, but not all. The test set contains three sequences with moving camera. Longer sequences were shortened to 1 minute in order to avoid individual videos from dominating the resulting evaluation scores. The dataset is freely available for download upon signing a Data Usage Agreement (DUA). The annotations are available at https://github.com/wosdetc/challenge, accessed on: 14 April 2021.

### 2.2. Evaluation Protocol

The Drone vs. Bird Detection Challenge 2020 allowed up to three submissions for each participating team. Each submission had to include a result file for each sequence similar to the provided annotation files. The particular result files had to provide the frame numbers and the predicted bounding boxes ([top_x, top_y, width, height]) and corresponding confidence scores for each frame. If the frame number was not listed, no detection was assumed. To rank the submitted results, the popular Average Precision (AP) was employed as evaluation metric. AP is based on the Intersection over Union (IoU) criterion for matching ground truth and detected object boxes. The IoU is the ratio of the area of overlap between the estimated bounding box and the ground truth bounding box to the total area of union:

$$IoU=\frac{\mathrm{detection}\cap \mathrm{ground}\;\mathrm{truth}}{\mathrm{detection}\cup \mathrm{ground}\;\mathrm{truth}}.$$

Detections are counted as true positive detections, if an IoU with a ground truth annotation is above 0.5. Otherwise detections are counted as false positives. Ground truth annotations that are not assigned to a detection, are counted as false negatives, i.e., missed detections. As the AP is calculated by taking the area under the precision-recall curve, it summarizes the whole precision-recall curve into a single metric and thus, encompasses the various precision-recall trade-offs of a detector. Note that the test sequences have been made publicly available one week before the submission deadline. Notice also that the rules of the challenge do not restrict the training data used by the algorithms to the Drone vs. Bird training set only.

### 2.3. Participation

**Figure 5:**
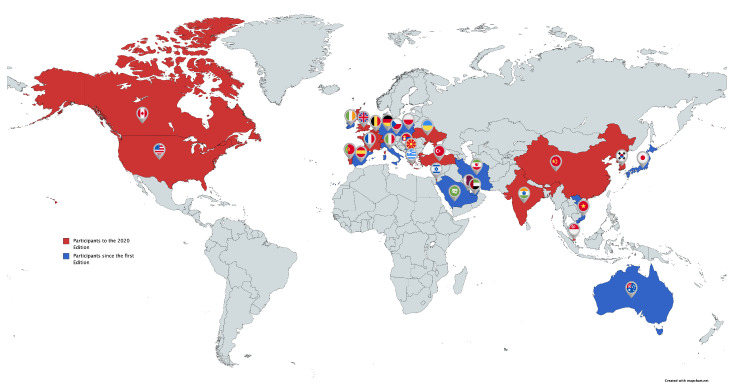
Research groups that participated to the Drone vs. Bird Challenge up to March 2021.

In this edition of the challenge, 23 teams from different countries requested the dataset, but only three of them succeeded in submitting valid results. In Figure 5, an overview of the nationalities of the teams that participated in the 2020 edition of the Drone vs. Bird challenge (marked with red color) is given, together with a more general overview of all the 108 research groups who requested the challenge dataset since its first edition (marked with blue color).

As concerns the relationship between the authors and the challenge, one should note that the first six co-authors of this paper organized and handled the challenge, including the analysis of the results submitted by the participating teams. The remaining co-authors are from the three teams that succeeded in submitting valid results.

## 3. Related Work

### 3.1. Drone Datasets

In the following, an overview of existing drone detection datasets that are publicly available is provided. Note that only datasets are reported that comprise EO or IR imagery, while drone detection datasets based on other sensor modalities are not considered.

The Drone Dataset: Amateur Unmanned Air Vehicle Detection [18] published in 2019 comprises more than 4000 images with drones, whereof the most images contain a DJI Phantom. The image resolution varies between 300 × 168 pixels and 4k. In addition, the dataset contains images with non-drone objects.

The Small Target Detection database (USC-GRAD-STDdb) [19] comprises 115 video segments retrieved from YouTube. The image resolution is 1280 × 720 pixels and annotations are provided for more than 25,000 frames. In total, more than 56,000 small objects whose sizes are in the range between 4 × 4 and 16 × 16 pixels are annotated. The small objects are categorized into drone, bird, boat, vehicle and person. 57 video segments with 12,139 frames contain either drones or birds.

The Purdue UAV dataset [20] consists of 5 video sequences of 1829 frames. The video sequences are recorded by a camera mounted on a custom airframe with 30 frames per second. The image resolution is either 1920 × 1080 or 1280 × 960 pixels. Each video sequence comprises up to 4 drones. Ground truth annotations for each video sequence are publicly available.

The Flying Object Detection from a Single Moving Camera dataset [21] comprises 20 video sequences with UAVs. Each image has a resolution of 752 × 480 pixels and contains up to two objects of the same model. The video sequences are recorded by a camera mounted on a flying drone. The variable appearance of drones due to changing attitudes, lighting conditions, and even aliasing and saturation caused by small sizes makes the dataset challenging. In addition, 20 video sequences with aircraft retrieved from Youtube are provided. The image resolution of these video sequences varies between 640 × 480 to 1280 × 720 pixels.

Recently, the Real World Object Detection Dataset for Quadcopter Unmanned Aerial Vehicle Detection [22] has been published. The dataset consists 51446 images for training and 5375 images for testing. The images are either downloaded from the Internet or recorded by the authors. As the original image resolution is in the range between 640 × 480 pixels and 4 k, all images are scaled to 640 × 480 pixels. The train set comprises 52,676 drone instances, while the test set only contains 2863 drone instances, so that 2750 images of the test set do not contain any drone. A semi-automated labelling pipeline has been used to speed-up the annotation task. In the train set, about 40.8% of drones are smaller than 32^2^ pixels, while 23.4% are larger than 96^2^ pixels. In the test set, approximately 36.3% of drones are smaller than 32^2^ pixels, while 28.3% are larger than 96^2^ pixels.

The Anti-UAV Challenge dataset [23] has been published in 2020. The 1st Anti-UAV Challenge was part of the of the IEEE Conference on Computer Vision and Pattern Recognition (CVPR) 2020. In contrast to the Drone vs. Bird Detection Challenge, the task of the Anti-UAV Challenge is single object tracking. The dataset comprises 160 video sequences (both IR and EO). The resolution of the IR images is 640 × 512 pixels and the resolution of the EO images is 1920 × 1080 pixels. Annotations are provided for 100 video sequences to train the tracking algorithms, while only the bounding boxes are given for the first frame of each test video. The videos are recorded by a static ground camera with an automatic rotation platform that can be remotely controlled by a computer. Hence, the image content is limited to only a few scenarios. Furthermore, only 4 different drone types are considered, i.e., DJI Inspire, DJI Phantom, DJI MavicAir and DJI MavicPRO.

The Multi-view drone tracking datasets [24], which has been proposed for reconstruction of 3D flight trajectories from ad-hoc camera networks, consist of five datasets. The first four datasets comprise a flying drone (hexacopter), which is captured with multiple consumer-grade cameras, while the latter one comprises 3 drones. The number of cameras ranges from 4 to 7 and the flight duration from 2 to 10 minutes. 2D annotations are provided for the first four datasets in terms of a single point. Compared to the Drone vs. Bird Detection Challenge dataset, these datasets are either smaller in size or diversity or designed for different tasks such as single object tracking or reconstruction of 3D trajectories.

### 3.2. Drone Detection

In recent years, the application of deep learning based detection methods led to excellent results for a wide range of applications including drone detection. Due to the absence of large amounts of drone detection datasets, a two-staged detection strategy has been proposed in [25]. The authors examined the suitability of different flying object detection techniques, i.e., frame differencing and background subtraction techniques, locally adaptive change detection and object proposal techniques [26], to extract region candidates in video data from static and moving cameras. In the second stage, a small CNN classification network is applied to distinguish each candidate region into the categories drone and clutter. In [27], a detection framework able to cope with targets of different size (in the range of less than ten to hundreds of pixels) as well as with varying illumination conditions has been proposed. Similar to [25], the approach consists of a first module used to detect the most probable regions containing the target, followed by a second module that classifies the detected target in either drone or other flying entity (e.g., a bird). The first module adopts the Region Proposal Network [28]—a deep learning based object proposal technique—to extract candidate regions, which are classified in the subsequent module by applying a CNN classifier. Spatio-temporal semantic segmentation has been exploited in [29] to detect small targets based on two separate CNNs. In a first step, a U-Net architecture is used to identify the areas of interest in an up-scaled version of the input image [30]. Then, the classification step is performed by resorting to a ResNet network, which selects the most probable areas containing the target of interest. The entire process is further enhanced by using spatio-temporal patches that represent typical drones trajectories; in doing so, the detector can more easily distinguish drones from birds. The adoption of different CNN architectures (e.g., Zeiler-Fergus (ZF) [31], Visual Geometry Group (VGG16) [32]) for drone detection has been investigated in [33]. To overcome the limited amount of data available for training the deep networks, authors exploited transfer learning from ImageNet and performed a pre-training to fine-tune the models. The experimental results revealed that VGG16 with Faster Region Based Convolutional Neural Network (R-CNN) achieves the best performance among all the considered architectures. The authors of Drone Detection in Long-Range Surveillance [34] worked on a previous iteration of the same dataset, with quite good results in the detection of small objects. They applied a Faster R-CNN network with various backbones and showed that ResNet-101 had the best results. In [35], an end-to-end detection algorithm able to predict the presence of a target in a sequence of video frames is presented. It is based on the adoption of the deep learning based detection method termed YOLOv2 [36], whose training is performed using an artificial dataset obtained by mixing real birds and drones images, each with a different background. The obtained results demonstrate that the diversity and the scale of the dataset have a positive impact on the detection and tracking processes. In [37], the authors adopted YOLOv2 for drone detection in images of a multi-camera array, while Unlu et al. [38] proposed the usage of its more recent variant YOLOv3 [39] for drone detection in a tracking pipeline. The applicability of the Single Shot MultiBox Detector (SSD) in an image processing pipeline for long range drone detection has been demonstrated in [12]. In [40], Super-Resolution (SR) techniques have been incorporated in the detection pipeline, with the aim of increasing the maximum detection range of the system. Before applying the detection algorithm, the input image is up-scaled by a factor of 2 by means of a deep DCSCN model [41]. A significant increase in the recall performance was observed by exploiting the joint optimization effects due to an end-to-end training of the models.

## 4. Gradiant Team

This section shows how to take advantage of CNN anchor based architectures by adapting these model parameters to characteristics of the bounding boxes present in the data, showing important improvements on accuracy. Notice that the results provided in this section do not refer to the final challenge test set and are not the ones used to compare the approaches from the different teams.

### 4.1. Methodology

The choice of Cascade R-CNN [42] architecture with a 101 ResNeXt as backbone was made from previous experience with Cascade and its performance in COCO benchmark. Furthermore, this model uses a FPN which adjusts to small drone detection, since its base level is constructed over a stride four with respect to the original image input size. This fact increases the capabilities of the network for detecting smaller drones compared to other popular architectures, as for example, RetinaNet [43] which uses stride 8 for the base level, at the cost of increasing computational complexity.

**Figure 6:**
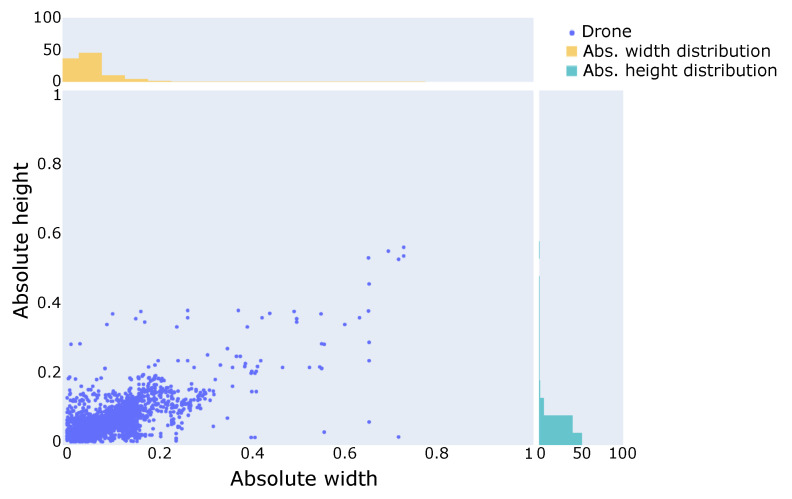
Dimension distribution for bounding boxes in the training set.

The main alteration made by Gradiant team was to design a reduced set of anchors that fit train data bounding box distribution so that the model can detect drones at very different scales with high accuracy. In localization based on bounding box regression, ground truth boxes only influence training loss when their maximum overlap with predefined anchors is greater than a specific threshold, usually predefined at 0.5. For this to happen, scales and aspect ratios of anchors have to be representative of the ground truth data distribution. In Figure 6 a visualization of training bounding box distribution in absolute pixels with respect to crop dimensions is shown. Two main facts can be observed from this plot: most boxes have small dimensions with respect to the crop size, and they tend to have larger width than height.

A new method that adjusts these anchors dynamically given the input image size, the number of feature pyramid levels and their correspondent strides, is proposed. The idea is to maximize the overlap between ground truth bounding boxes and anchors, trying to match all boxes with at least one anchor by making the overlap between them greater than the established threshold. The authors have open sourced all code and made it publicly available in Github https://github.com/Gradiant/pyodi, accessed on: 14 April 2021.

**Figure 7:**
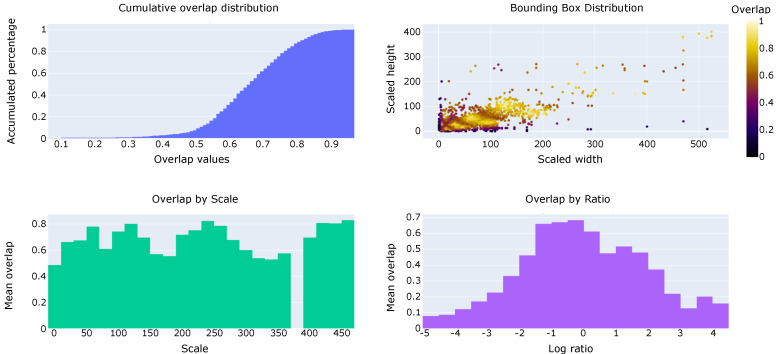
Results for anchor matching.

Figure 7 shows four different plots that summarize the result of this matching. In the upper left corner it can be observed a cumulative overlap distribution over the matching results. Less than a 10% of the total bounding boxes have maximum overlap values below 0.5, this is, only less than a 10% ground truth bounding boxes will not influence the training (when the augmentation effects are not taken into account). Upper right corners show the distribution of the bounding boxes dimension, color intensity represents the maximum overlap that each of them obtain after matching with generated anchors. Most low matches, represented with dark colors, are produced for extreme scale ratios. Finally, two bottom plots represent an histogram with relation between bounding scale and mean overlap (left) and log scaled scale ratios.

### 4.2. Datasets Used

In order to ensure a robust validation strategy, the authors manually split the videos in the train and validation data trying to maintain both sets as independent as possible. Hence, videos that were recorded at exactly the same location must belong to the same data split. A final selection of 16 videos was used as validation set.

Most videos were recorded at high frame per second rates which results in a large amount of identical images. For avoiding this effect a subsampling was carried on by taking one out of each ten frames, in other words, only a 10% out of the total data was used for training and validating our model.

Apart from challenge data, external datasets were used for training the final model. The first of them is the Purdue UAV dataset [20], which can be publicly accessed and contains a total of 70,250 frames that were also subsampled until reaching 27,864 frames with 10,461 bounding boxes. The second dataset is private and consists of 22,236 frames and 14,152 bounding boxes.

Most of the images have a resolution of 1980 × 1080 pixels and training a network with images of such dimensions is unmanageable. The effect of downscaling input images would result in a loss of information, which is critical for detecting very small drones. Areas of small objects would embrace only a few pixels after being resized; for this reason, image crops of 720 × 720 pixels were extracted from each image, following a sliding windows approach.

Furthermore, different techniques for data augmentation were applied such as the addition of different types of noise, such as Gaussian or Median blur. Color transformations such as RGB shift, HSV shift, Additive Random Brightness and other transformations (e.g., random rotations or random cutouts) were also performed. All these operations were added in a stochastic fashion during the training phase so images in each data batch were randomly altered.

### 4.3. Experimental Evaluation

In order to evaluate the performance of the proposed approach, a comparison between three different configurations is provided. The first of them uses a standard Cascade R-CNN architecture, with standard anchor configuration extracted from COCO dataset and has been trained using all previously mentioned data sources. The second model uses custom anchors and has been trained using the Drone vs. Bird challenge data only. Finally, the model submitted to the grand challenge uses personalized anchors and has been trained with all the available data.

**Figure 8:**
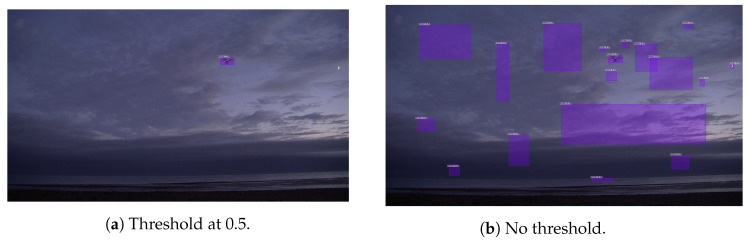
Comparison between different thresholds under same image.

All experiment results have been obtained over the previously mentioned validation dataset which consist of 16 videos. Although competition results are evaluated against AP@0.5, this metric is not useful for evaluating how a model would perform in real world [44]. In such situations, a confidence threshold for discarding low quality predictions and fusing its result with other sensors must be provided. All these facts are not taken into account with AP which suggests the use of other metrics that better align with real applications. In addition, most datasets for drone detection include very few categories (usually one) and often just an object per image. Under these settings, the AP ignores all false positives as long as the prediction with the higher confidence is assigned to the object of interest, as illustrated in Figure 8, where both images obtain the same AP (1.0–1.0) but a huge gap in moLRP (0.0–0.944).

**Figure 9:**
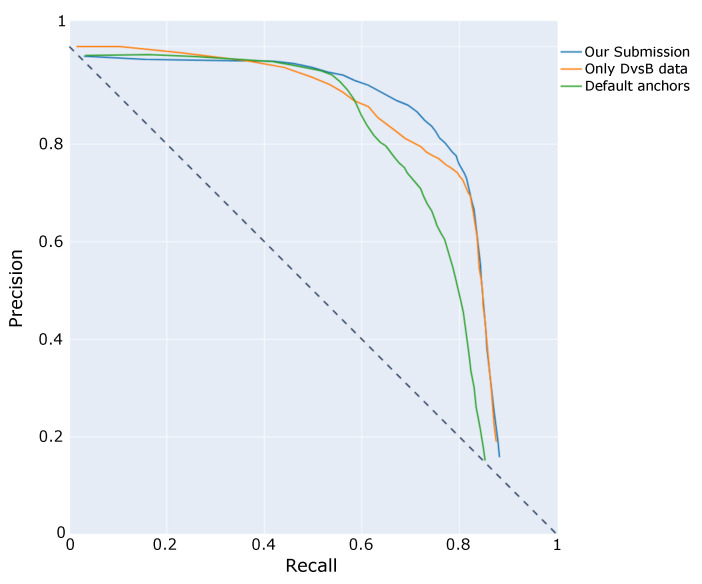
Precision vs. Recall at IoU = 0.5.

Figure 9 reports a precision versus recall at IoU score of 0.5 comparison of the three different models. It can be easily observed how default anchors model has the worst performance, which shows the importance of designing a proper anchor set for object detection problems like the one in this challenge. Differences between final submission and the model trained only with a reduced dataset are highlighted when model threshold is increased, whereas the final submission is able to maintain higher recall values.

**Figure 10:**
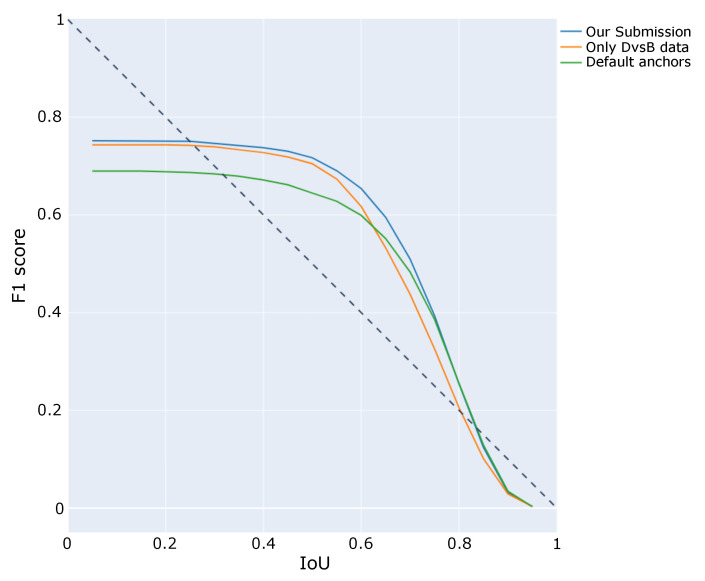
F1 score result for different IoU thresholds.

A similar comparison is shown in Figure 10 where different IoU values and their corresponding F1-score values are plotted for each of the trained models. Once again, best results are achieved by the submitted model. For larger IoU values the three of them converge to similar scores and this is due to the difficulty of achieving large IoU values for very small ground truth objects, where small differences result in large changes in IoU.

**Figure 11:**
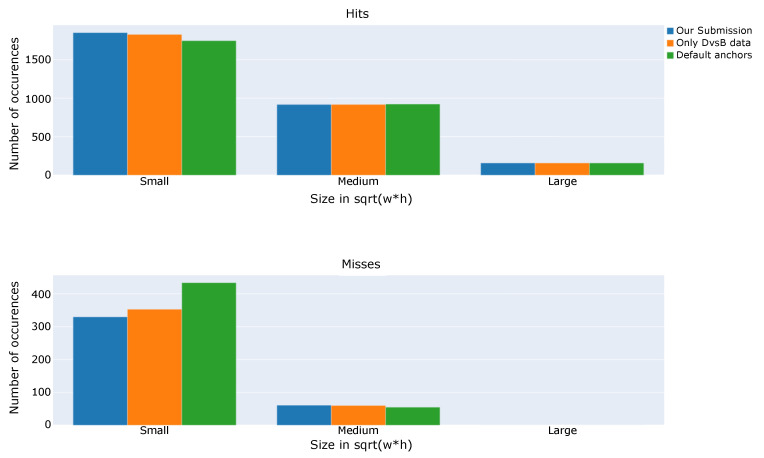
Hits and misses for different bounding box scales.

In Figure 11, a comparison between the number of hits (true positives) and misses (false negatives) is shown. Ground truth bounding boxes are divided in three different categories depending on their scale, so that one can visualize how models perform with respect to target sizes. It is clear that main differences occur for small drone detection, where models trained with our custom anchor set are able to achieve a higher number of hits and at the same time reduce the number of misses. This fact supports the decision made by Gradiant Team of creating specific anchors suitable to match small drones during training and increase the capabilities of the model for detecting them.

### 4.4. Discussion

The proposed method by Gradiant Team focuses on reducing the gap between model architecture and dataset characteristics. Factors such the characteristic shapes of drones, their size and their position on the image can be used to optimize training. The use of dynamic anchors extracted from the datasets outperforms models that use traditional anchor sets that were designed for working in general object detection problems. It is clear that main differences occur for small drones, where custom anchors boost detection capabilities of the model. Nevertheless, further progresses will require the use of temporal information to integrate frame information and help, for instance, to characterize those drones that move away from the camera. Furthermore, the exploration of new metrics that properly represent the complexity of this challenge must be addressed, in order to reinforce the exploration of techniques and models that can be used in real environments.

## 5. EagleDrone Team

The approach proposed by Mitra and Rajashekar (referred to as EagleDrone team) adopts pyramid networks combined with Faster R-CNN. Issues with training using less data and lower resolution impacts of the images are also investigated. As for the previous section, the results provided here do not refer to the final challenge test set.

### 5.1. Methodology

In some detection tasks, accuracy of the detection model is in higher priority compared to speed. Two-stage detectors have a better accuracy but a slower inference time. Faster R-CNN was indeed selected for its best speed. Real-time surveillance videos show that birds or drones cannot move into the camera view angle and disappear from the frame within a few seconds. Faster R-CNN has an inference time of 0.2 s per frame so even if few frames are missed, accurate advisories can be provided. Since birds and drones are quite small in the videos, a feature pyramid network was introduced in addition to the Faster R-CNN.

Additionally to Faster R-CNN, YOLOv5 was tested to evaluate the balance between speed and performance. Moreover, the use of super-resolution techniques to improve performance was explored, using ESRGAN [45].

### 5.2. Datasets Used

There were three open-sourced datasets which were considered for the project. These included Little Birds in Aerial Images, Drone vs. Bird Competition dataset, and Birds in background of Windmills dataset.

**Figure 12:**
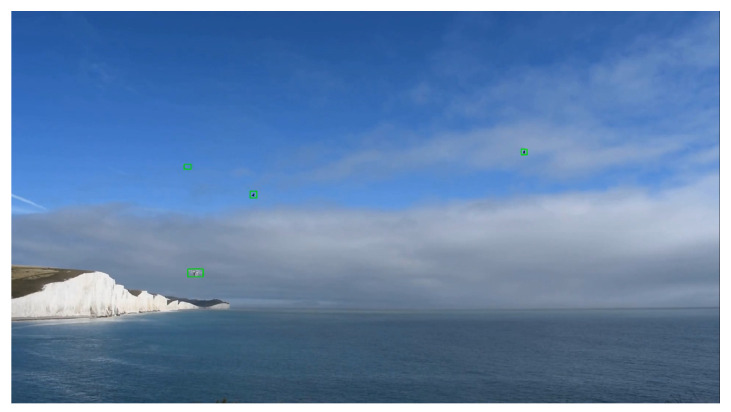
Detection of Multiple Objects.

The first dataset had images from an aerial view. As the problem statement required images ground-level view, the first dataset could not be used. The other two datasets had problems with annotations. The second one, which is provided for the challenge, had annotations for only drones while the last dataset had annotations for birds. In the third plausible dataset, the background and the position of the camera was always fixed. As it is preferable to choose a dataset with enough variation, only the Drone vs. Bird challenge dataset was selected, since it had a good number of videos with different shapes of drones and backgrounds, and no additional dataset was included in the training of the algorithm. Figure 12 is an example that shows multiple drones in the same image.

### 5.3. Experimental Evaluation

An initial training step was done on a GPU in Google Colab. Due to file constraints on Google Colab, only 3000 images were chosen for the first iteration of the training. 150 images were chosen for the validation set. Training was done for 30 epochs and the validation loss was monitored. The checkpoints where the validation loss decreased was saved and it ensured that the saved checkpoint was not overfitting. The training loss decreased as expected and validation score was close to the training score and the nature of the curve indicated that overfitting had not started till the last few epochs.

**Figure 13:**
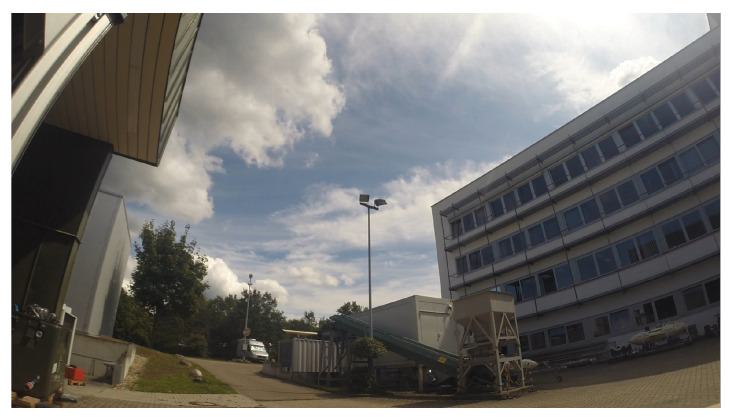
Example of drone detection issue.

**Figure 14:**
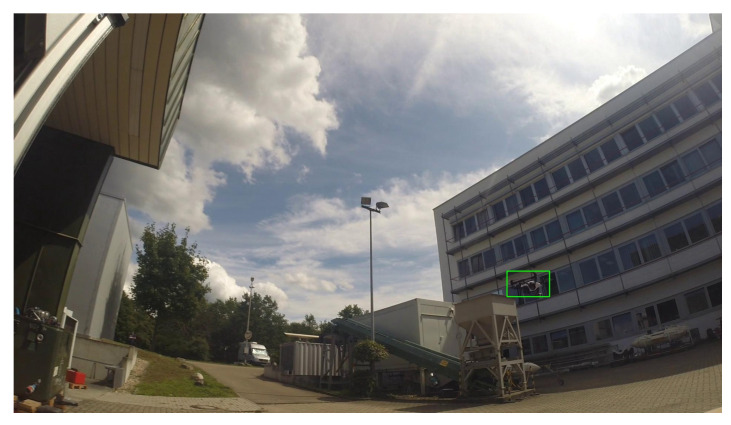
Drone detection is feasible in white and blue background.

After the first round of training, it was seen that in images like Figure 13, where the background does not contain the sky, the drone was not detected. Figure 14 contains blue and white background and the model can easily detect the drone. This is due to the fact that in the training images, there were mostly objects with sky as the background so the model had considered that fact to classify the object. Also, it classified birds as drones. This might be because training images with birds were very low and contained drones that looked like birds. Thus the model could not understand the fact that those were a separate class.

To address this problem, the loss on all the images were calculated. Images with objects being unclassified will have a higher loss and this would have given an idea of which images contain the most unclassified objects. 3000 of such images were selected using a linear sampling probability. Thus the one having the higher error had the greater chance of selection. It was retrained using the same method as before.

**Figure 15:**
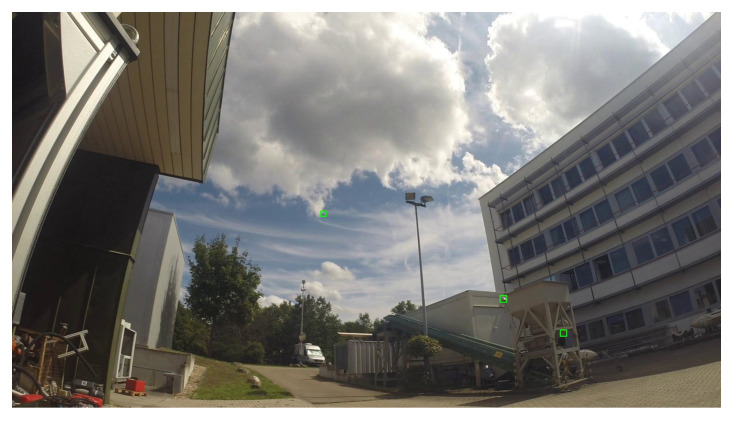
Model classifying random objects.

This variation of hard modelling removed the previous problem but it unclassified other things as seen in Figure 15 [46]. Measuring Catastrophic Forgetting in Neural Networks might be a reason for why this was happening. When the model received the new images, it fine tuned the weights to predict the objects in the new set of images. But as a result of the fine tuning, the model did not store the information that was present before fine tuning.

**Figure 16:**
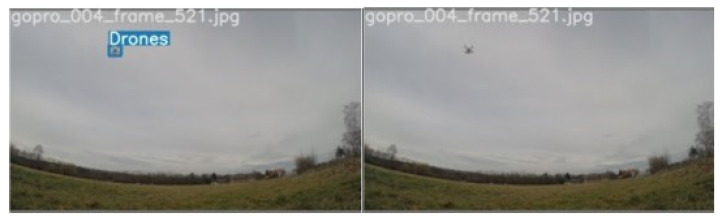
Left image is the ground truth. In the right image, YOLOv5 is not able to detect.

YOLOv5 is the best one stage object detector that is available at the moment. The higher models could not be fit into the Colab, so YOLOv5s which is the smallest of the models in their model base was tried. Figure 16 shows that YOLOv5 did not give excellent results and as it was unable to perform, further investigation on one-stage object detectors was not done.

**Figure 17:**
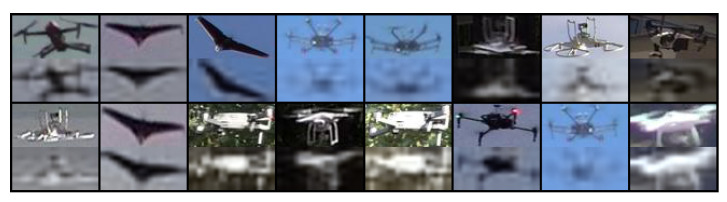
Examples of ESRGAN in given Dataset. The top row indicates original image, the bottom indicates respective GAN images.

ESRGAN is one of the most successful super-resolution technique available. In this model, a Generative Adversarial Network is used. The generator is a model that generates a super-resolution image from a low-resolution image. The discriminator tries to predict if the image generated is a super-resolution image or a high-resolution image. The generator tries to fool the discriminator while the discriminator helps the generator to improve by letting it know where changes need to be done. The authors chose 80 × 40 resolution images and downsized them to 20 × 10 images and tried to bring them back to 80 × 40. The results were not promising, as it can be observed in Figure 17, although the approach performed better than normal bi-linear interpolation. In the original paper, the image sizes were 128 × 128 and they were extended to 512 × 512. In this case, the low-resolution image size are about one-eighth of the original size. The 20 × 10 image quality was already degraded, hence applying this technique was not beneficial.

### 5.4. Discussion

Due to the limitations of Google Colab, all the images (95,000) could not be trained at once. Future enhancement could be to train the complete dataset in a single experiment using a high-end machine. The super-resolution technique can be added to the model so that it helps to classify smaller objects. Video object detection can also be investigated so that classification between birds and drones can be easily done. Multi-sensory data from radar and other sources can also be used to further improve on the model.

## 6. Alexis Team

Gagné and Mercier (referred to as Alexis team) proposed a drone detection approach based on YOLOv3 [39] and taking a single RGB frame as input. By integrating an image tiling strategy, this approach is able to successfully detect small drones in high resolution images. In addition, the tiling method can reduce the processing cost to track a drone. After the first detection in the entire frame, the algorithm can remain focused on the region of interest around the target and reprocess the entire frame every X frames to detect new drones. Also in this case, the results provided in this section do not refer to the final challenge test set.

### 6.1. Methodology

Alexis team selected the single-stage object detector YOLOv3 [39] because of its good trade-off between speed and accuracy. In this section, its architecture, implementation, and training are discussed.

In YOLOv3, predictions are made at 3 different scales (1/32, 1/16 and 1/8 of the input image resolution). For each scale, it uses 3 predefined anchors for which they selected the default sizes of (10 × 13), (16 × 30), (33 × 23), (30 × 61), (62 × 45), (59 × 119), (116 × 90), (156 × 198) and (373 × 326) pixels. For each anchor, the network regresses the bounding box location/dimensions and predicts an objectness score (class-agnostic) along with class-specific scores.

Alexis team leveraged the public PyTorch implementation of YOLOv3 with Spatial Pyramid Pooling (YOLOv3-SPP) made available by Ultralytics [47]. Spatial Pyramid Pooling [48] is a simple technique for which the input features are processed by pooling layers of different sizes in parallel and then concatenated to generate fixed-length feature vectors. For SPP employed in combination with YOLO [49], the original feature map resolution is kept by applying max pooling with appropriate padding. Output feature maps are then all concatenated together along with the input. In YOLOv3-SPP, the SPP layer is added between the end of the backbone and the YOLO classification/regression layers.

**Figure 18:**
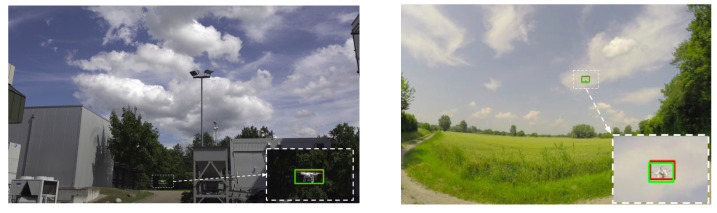
Alexis team approach based on YOLOv3 [39] is able to detect small drones in high resolution images by using an image tiling strategy. Here are some examples of successful detections made by their approach (green: predictions, red: ground-truths).

Alexis team used YOLOv3-SPP pretrained on the Microsoft Coco dataset [50]. Then, the network was trained with Stochastic Gradient Descent (SGD) for 5 epochs with a learning rate of 0.001 (multiplied by a factor of 0.5 after epochs 3 and 4) and mini-batches of 24 images (when using a training resolution of 480px). The network was trained using a single class (drone) and was optimized for objectness (using the binary cross-entropy loss) and bounding box regression (using the complete Intersection loss [51] instead of the sum of squared error loss proposed in YOLOv3 [39]). Figure 18 shows examples of successful detections made by Alexis team approach.

### 6.2. Datasets Used

In this section, the datasets used for training is described, along with the strategy for image tiling and data augmentation.

The main dataset to train and test their approach was the 2020 Drone vs. Bird Challenge. Alexis team removed one of the 77 available annotated sequence (it appeared to have bad annotations) for a total of 106,485 frames. The dataset contains instances of drones and birds, but is annotated for drones only.

Additionally, Alexis team generated 26,500 synthetic images following the procedure suggested in the paper *Cut, Paste and Learn* [52]. In order to generate the dataset, around 100 close-up images of drones and birds were acquired from Google Images. Object masks were manually generated. The objects were then pasted on outdoor backgrounds using different blending methods. An example of such image is shown in Figure 21a. Similarly to the Drone vs. Bird dataset, bounding box annotations were used only for drones.

**Figure 19:**
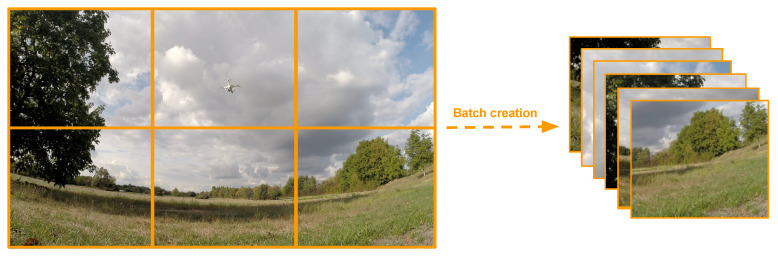
Image tiling strategy used at test time. The image is divided into multiple tiles of a certain size. These tiles are then concatenated to form a batch, which is given in input to the detection network. Predictions from each tiles are aggregated by adding offsets corresponding to their location in the original image. In their implementation, overlaps between tiles are added for better robustness.

The particularity of drone detection is that drones can be really small (10–20 pixels) compared to a full high-resolution image. The standard procedure of downsizing images before feeding them to deep convolutional neural networks is therefore impractical, as drones can become too small to be detected or distinguished from other flying objects. At the cost of higher processing times, image tiling [53] is a strategy that has shown great performances for detecting small objects. As shown in Figure 19, the image tiling strategy consists of dividing the input image into multiple patches/tiles. For more robustness and redundancy, an overlap can be added between tiles. It can especially help in cases where drones are at the border of a tile.

**Figure 20:**
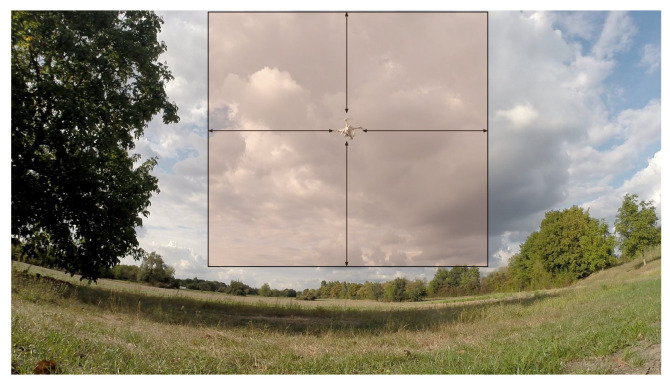
Random cropping of an image patch/tile during training. In most cases (80% of the time), the crop location is sampled to contain at least one drone. The rectangle in the image shows the area covered by the possible crop locations for this drone.

During training, Alexis team simulated image tiling with random cropping. In 80% of the cases, a crop location is sampled that contained at least one drone (as shown in Figure 20). Otherwise, the crop location is completely random. This strategy helps in showing more positive examples of drones while also showing background examples.

**Figure 21:**
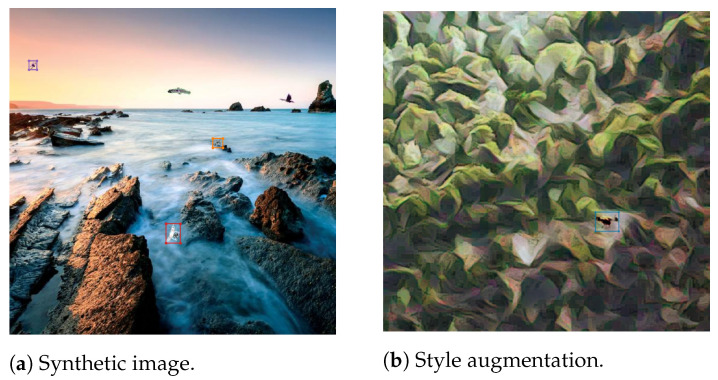
Examples of different augmentations. In (**a**), this is a synthetic image generated with Alexis team pipeline and (**b**) is an image on which style augmentation [55] has been applied.

During training, data augmentation techniques such as affine transformations, flips, blurring, noise, brightness and several other augmentations from the Albumentations package [54] were employed. Also, Alexis team used style augmentation [55], which is based on style transfer and has been shown to improve robustness to domain shifts. An example of an image for which style augmentation has been applied to is shown in Figure 21b.

### 6.3. Experimental Evaluation

In this section, the performance of the approach is evaluated and experiments are reported to show the impact of some hyperparameters such as the tile size and data selection and augmentation strategies, which can have a significant impact. The AP was used, which is the standard metric for object detection, for the evaluation.

In the following experiments, two different data sampling strategies to better measure the generalization performance of their approach were used. The first data sampling strategy used is the standard way of splitting data: all images were sampled into training (85% of the images), validation (5%) and test (10%) sets. This strategy was named “known sequences”, since images were sampled without taking the video sequence of the Drone vs. Bird dataset into account (meaning that images in the test set could come from the same sequence as images of the training set). For the second strategy, which is called “unknown sequences”, contain exclusively 12 selected sequences for the test set and the images from the remaining sequences were selected for training and validation. This is a more realistic way of measuring the generalization performance of the approach by decreasing the odds of overfitting the test sequences.

Alexis team first evaluated the impact of tile resolution on a seen and unseen sequence. Results of this experiment are reported in Table 1. From the results, a tile resolution of 480 pixels seems to be a good compromise in terms of detection performances and processing time. The performance was evaluated on a PC equipped with an Intel Core i7-7820X CPU, a Samsung 970 EVO PLUS SSD and a NVIDIA GeForce RTX 2080 Ti GPU.

Subsequently, the impact of employing synthetic data and style augmentation during training was evaluated. The results in Table 2 are reported for a test set filled with images from an unseen sequence. In the first case, the performance of the approach when trained only with images from the Drone vs. Bird (DvB) Challenge was evaluated. The impact of adding the synthetic dataset and style augmentation was then evaluated. One can see that for that data split, both strategies helped this approach to generalize better to images of the unseen sequence.

Finally, the average performance for 5 different data splits for both seen and unseen sequences are reported in Table 3. For the experiments, a tile size of 480 pixels and an overlap of 100 pixels between each tile were used.

### 6.4. Discussion

In their approach, Alexis team proposed to use YOLOv3 and use an image tiling strategy to better detect small drones. To get a better generalization to different domains/settings, they additionally used synthetic images and data augmentation techniques such as style augmentation.

However, even with these strategies, due to the low background diversity in the dataset likely caused by physical constraints, there is a high likelihood that some parts of the dataset were overfitted. Results in Table 3 show the gap in performance when testing on sequences that were not seen during training. It might be something of interest to investigate in future iterations of the challenge.

Alexis team also noted that since their network is trained only to detect a single class (drones), their detector is biased towards detecting drones and can struggle to distinguish them from other similar-looking flying objects (especially if they are really small). Potential solutions to reduce that bias would be to either train for more object classes (acquire more images containing objects such as birds, planes, helicopters, etc. and annotate them) or use multiple frames to acquire information about their flying patterns. For this project, they have attempted to train with more negative examples (without annotating them). They were able to decrease the number of false detections using this strategy. However, it also significantly decreased the number of good detections, which resulted in worse overall performance of the detector.

Finally, for the challenge, Alexis team submitted two different versions of their approach. The first one was trained with a combination of the Drone vs. Bird training dataset and their synthetic images and the second one did not use the synthetic images. While training with synthetic images improved results by 3% in own evaluation (Table 2), it resulted in only a 0.2% improvement in the official challenge. A possible explanation can be that the challenge was easier for their approach than the unseen data split they evaluated on, therefore limiting the impact of the synthetic images.

## 7. Performance Comparison

This section discusses the results using the final challenge test set, in order to compare the approaches from the different teams. The Drone vs. Bird Detection Challenge 2020 allowed up to three submissions for each participating team. Submission results for the top three teams are given in Table 4. The evaluation relies on the popular AP metric for object detection. Detections are counted as correct, when its intersection over union (IoU) with a ground truth box is above 0.5. The final resulting scores are averaged across all 14 sequences of the test set.

Team Gradiant obtained the best overall performance of 80% with Cascade R-CNN [42] applied on 720 pixels crops using a sliding windows approach. In run 1, they personalized the anchors and trained on additional datasets. In run 2, they trained with the standard anchors while still training with additional data and in run 3, they used their personalized anchors, but only trained with the data from this challenge. Team Alexis finished in second place. They used YOLOv3 [39] applied on image crops of 480 pixels. In run 1, they used additional synthetic data during training whereas in run 2, they only used the training data of the challenge. Team Eagledrone finished in 3rd place by using Faster-RCNN [28]. Due to limitations, they only trained their model on a subset of 3000 images.

**Figure 22:**
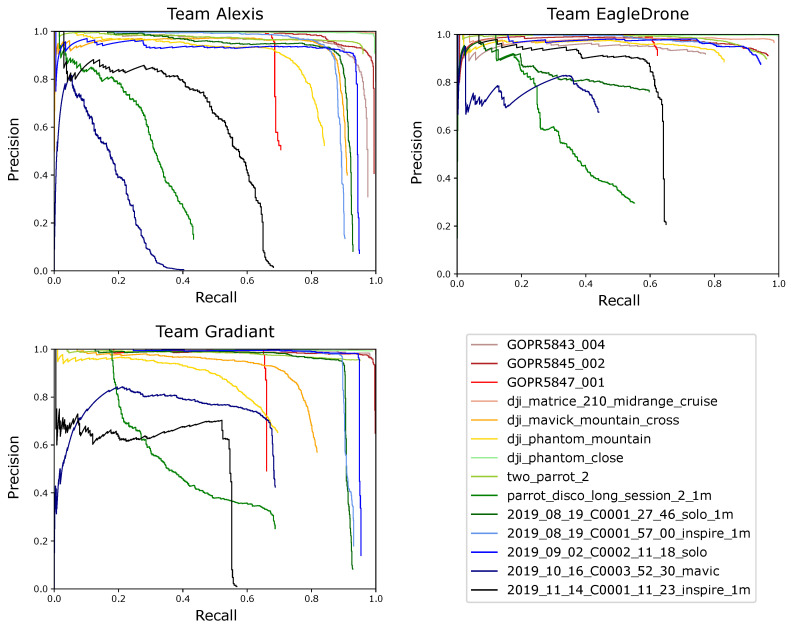
Comparison of the detection results in terms of precision-recall curves.

Table 5 and Table 6 list the resulting scores of each teams’ best submission in more detail for each of the test sequences separately. In addition, precision-recall curves given in Figure 22 are used to visualize the resulting scores of each teams’ best submission for each of the test sequences.

Table 5 clearly shows that there is a range in difficulty among the given test sequences. Some sequences (e.g., dji_matrice_210_midrange_cruise) can be solved almost perfectly, due to a relatively large and clearly visible drone in the sequence. Other sequences, however, are much more difficult to handle for all approaches. This is particularly the case for the latter two sequences that are either recorded by a moving camera or at twilight. But also sequences that contain segments with very distant drones (e.g., GOPR5847_001) can be challenging to the evaluated methods. Furthermore, the results show that the submitted approaches actually perform somewhat complementary. On several sequences the performances vary among the three approaches yet each approach achieves the best overall score on some of the sequences. A combination of the design aspects of the three methods thus looks to be a promising direction for future work.

Further insight into the resulting scores can be gained from Table 6, which compares the number of present ground truth objects to the number of submitted detections, resulting recall and implicitly the number of false detections. It can be observed that the Eagledrone team generated much fewer detections, resulting in fewer false positives but also negatively impacting the overall recall and resulting AP. Conversely, the other two teams’ results exhibit very large numbers of detections, and thus many false positives, for some of the test sequences. Naturally, such large numbers of detections result in better recall. The fact that also AP is comparatively good indicates that many of these false positive detections are of a very low confidence score and thus do not affect AP very strongly and could be filtered out in practical application.

**Figure 23:**
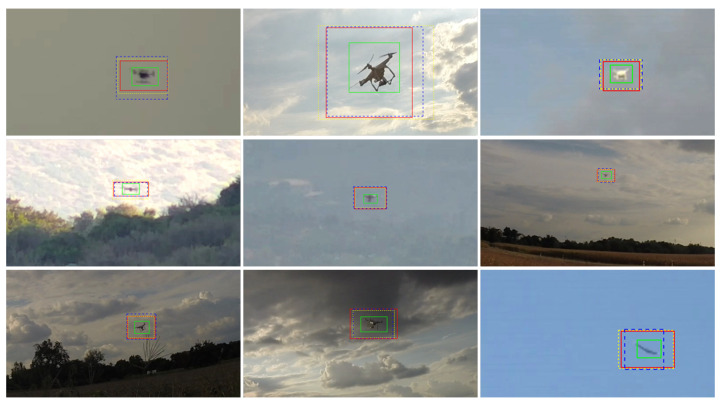
Images crops showing qualitative detection results and the corresponding GT (green). Note that the detection results from Team Alexis (red), Team Eagledrone (blue) and Team Gradient (yellow) are enlarged to simplify the visualization. All methods achieve good detection results for different drone types and a large range of drone sizes in different environments.

**Figure 24:**
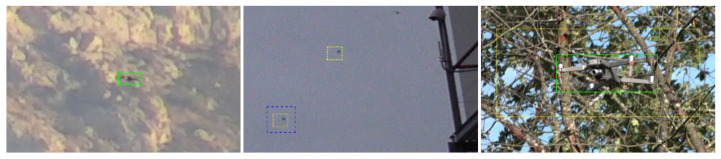
Images crops showing qualitative detection results and the corresponding GT (green). Note that the detection results from Team Alexis (red), Team Eagledrone (blue) and Team Gradient (yellow) are enlarged to simplify the visualization. Remaining challenges are mainly small drones in front of structured backgrounds, small disturbing objects and drones in front of unseen structured backgrounds.

Figure 23 and Figure 24 show qualitative detection results of each teams’ best submission. All methods achieve good detection results for different drone types and a large range of drone sizes in different environments (see Figure 23). Negative detection results given in Figure 24 indicate remaining challenges such as small drones in front of structured backgrounds, small disturbing objects and drones in front of unseen structured backgrounds.

## 8. Conclusions

This work presented the results of the 2020 Drone vs. Birds detection challenge. The three best performing approaches were described and compared. The methods differ in key design aspects and the results show that complementary strengths are exhibited for each of the approaches. One should observe that the key challenges lie in the handling of moving cameras and detection of distant drones. Thus, in future editions the training and testing data will be expanded along these characteristics. A promising methodological direction for future work will be to investigate the combination of design aspects from the three approaches in order to evaluate and exploit their complementary nature.

It is worth noticing that the developed algorithms do not explicitly take birds into account at the design stage in a supervised manner, since data are not annotated for birds. Consequently, in test sequences where several birds appear (in particular, in one scene an entire flock is present) false alarms generally increase for all methods, as birds are small as in case of distant UAVs. Annotation of video sequences also for birds is clearly an highly time-consuming task hence is not scalable when in scenarios where many birds are found. One of the tasks to be addressed is thus how to include bird targets in the training dataset, also considering that the appearance of distant fixed-wing UAVs is very similar to birds. Further research is needed on this topic, and will be considered in the future editions of the Drone vs. Bird Detection Challenge.

Another aspect that deserves investigation is the understanding and interpretation of the ultimate reasons why each algorithm has different performance on different sequences. Future editions of the challenge may evaluate additional aspects such as computational complexity (and real-time), use of additional datasets, ability to generalize to completely different backgrounds, etc., as a means to grasp understanding of the many different trade-offs at play among the numerous possible approaches and methodological combinations.

## Figures and Tables

**Table 1:** Impact of tile resolution on detection performances and processing time per image evaluated on images from a seen and an unseen sequence.

Metrics/Tile Size (px)	320	480	640
Known sequence AP (%)	91.3	92.9	89.1
Unknown sequence AP (%)	56.1	57.8	44.3
Processing time per image (ms)	195	169	125

**Table 2:** Performance of different training strategies evaluated on images from an unknown sequence. The performance is reported when training only with images from the Drone vs. Bird (DvB) Challenge and when the synthetic dataset and style augmentation are added.

Training	AP (%)
DvB	52.2
DvB + synthetic	55.6
DvB + synthetic + style augmentation	57.8

**Table 3:** Results (AP) of Alexis team approach when testing on known and unknown sequences sampled from the 2020 Drone vs. Birds Challenge. The mean and standard deviation for 5 different data splits (1 run per data split) was reported.

Sequence	AP (%)
Known	93.55 ± 0.6
Unknown	66.0 ± 13.5

**Table 4:** Comparison of detection results of the Drone vs. Bird Detection Challenge 2020. Each team was allowed up to three submissions.

Submission	Alexis [%]	Eagledrone [%]	Gradiant [%]
Run 1	79.8	66.8	**80.0**
Run 2	79.4	✗	72.9
Run 3	✗	✗	75.2

**Table 5:** Detailed comparison best run for each team in the Drone vs. Bird Detection Challenge 2020. The first column denotes static or moving camera. The evaluation metric used for this dataset is AP. The evaluation is given for every sequence of the test set. The overall averaged result is given at the bottom.

Sequence	Static	Alexis [%]	Eagledrone [%]	Gradiant [%]
GOPR5843_004	✓	94.3	74.1	**97.7**
GOPR5845_002	✓	98.1	94.7	98.7
GOPR5847_001	✓	69.6	62.2	65.7
dji_matrice_210_midrange_cruise	✓	99.8	97.1	99.9
dji_mavick_mountain_cross	✗	87.2	80.2	77.3
dji_phantom_mountain	✓	78.2	52.3	63.1
dji_phantom_close	✓	99.3	99.4	98.8
two_parrot_2	✓	93.7	95.0	93.5
parrot_disco_long_session_2_1m	✗	28.9	37.3	42.3
2019_08_19_C0001_27_46_solo_1m	✓	88.6	51.1	90.0
2019_08_19_C0001_57_00_inspire_1m	✓	87.9	81.0	91.2
2019_09_02_C0002_11_18_solo	✓	89.1	92.2	94.6
2019_10_16_C0003_52_30_mavic	✗	15.8	36.3	54.8
2019_11_14_C0001_11_23_inspire_1m	✓	49.3	59.9	38.9
Overall		79.8	66.8	80.0

**Table 6:** Comparison of detection results in terms of number of submitted detections (#Det) and resulting recall.

Sequence	#GT	Alexis		Eagledrone		Gradiant	
		#Det	Recall	#Det	Recall	#Det	Recall
GOPR5843_004	438	1381	97.5	368	77.2	447	98.2
GOPR5845_002	544	1330	99.4	577	96.7	836	99.8
GOPR5847_001	318	443	70.4	217	62.3	427	66.0
dji_matrice_210_midrange_cruise	1469	1853	99.9	1498	98.5	1499	99.9
dji_mavick_mountain_cross	1551	3528	91.1	1453	83.0	2223	81.8
dji_phantom_mountain	1663	2668	84.0	1245	56.2	1774	69.5
dji_phantom_close	1193	1307	99.6	1195	99.6	1188	98.9
two_parrot_2	2789	2951	96.0	2990	96.2	2783	95.1
parrot_disco_long_session_2_1m	789	2585	43.3	1479	55.1	2151	68.7
2019_08_19_C0001_27_46_solo_1m	926	10,625	92.9	727	59.7	10,394	92.9
2019_08_19_C0001_57_00_inspire_1m	1219	8178	90.4	1052	81.9	6338	93.1
2019_09_02_C0002_11_18_solo	626	8152	89.1	676	94.4	4284	95.4
2019_10_16_C0003_52_30_mavic	733	57,005	40.2	478	44.1	1188	68.8
2019_11_14_C0001_11_23_inspire_1m	368	16,928	68.2	1156	64.9	21,898	56.8
Overall	14,626	118,934	87.4	15,102	72.9	57,430	87.2

## Data Availability

The Drone-vs-Bird challenge dataset is freely available for download upon signing a DUA. The annotations are available at https://github.com/wosdetc/challenge, accessed on: 14 April 2021. Gradiant Team has open sourced all the code and made it available at https://github.com/Gradiant/pyodi, accessed on: 14 April 2021.
